# An outbreak investigation of scrub typhus in Western Province, Solomon Islands, 2014

**DOI:** 10.5365/WPSAR.2015.6.3.007

**Published:** 2016-01-26

**Authors:** Michael Marks, Cynthia Joshua, Jenny Longbottom, Katherine Longbottom, Alison Sio, Elliot Puiahi, Greg Jilini, John Stenos, Tenneth Dalipanda, Jennie Musto

**Affiliations:** a Clinical Research Department, Faculty of Tropical and Infectious Diseases, London School of Hygiene and Tropical Medicine, London, United Kingdom.; b Hospital for Tropical Diseases, University College London Hospitals NHS Trust, London, United Kingdom.; c Office of the WHO Representative in Solomon Islands, Honiara, Solomon Islands.; d Ministry of Health and Medical Services, Honiara, Solomon Islands.; e Helena Goldie Hospital, Munda, Solomon Islands.; f National Referral Hospital, Honiara, Solomon Islands.; g Australian Rickettsial Reference Laboratory, Geelong, Australia.

## Abstract

**Objective:**

To identify the etiology and risk factors of undifferentiated fever in a cluster of patients in Western Province, Solomon Islands, May 2014.

**Methods:**

An outbreak investigation with a case control study was conducted. A case was defined as an inpatient in one hospital in Western Province, Solomon Islands with high fever (> 38.5 °C) and a negative malaria microscopy test admitted between 1 and 31 May 2014. Asymptomatic controls matched with the cases residentially were recruited in a ratio of 1:2. Serum samples from the subjects were tested for rickettsial infections using indirect micro-immunofluorescence assay.

**Results:**

Nine cases met the outbreak case definition. All cases were male. An eschar was noted in five cases (55%), and one developed pneumonitis. We did not identify any environmental factors associated with illness. Serum samples of all five follow-up cases (100%) had strong-positive IgG responses to scrub typhus. Nine out of ten controls were negative for ST antibodies. Four controls had low levels of antibodies against spotted fever group rickettsia, and only one had a low-level response to typhus group rickettsia.

**Discussion:**

This outbreak represents the first laboratory-confirmed outbreak of scrub typhus in the Western Province of Solomon Islands. The results suggest that rickettsial infections are more common than currently recognized as a cause of an acute febrile illness. A revised clinical case definition for rickettsial infections and treatment guidelines were developed and shared with provincial health staff for better surveillance and response to future outbreaks of a similar kind.

## Introduction

Rickettsial infections classically present as an undifferentiated fever syndrome. Rash, eschars and lymphadenopathy occur at varying frequencies depending on the causative organism. (*1*) Scrub typhus, caused by *Orientia tsutsugamushi*, is spread by larval (chigger) trombiculid mites from a limited range of species. *O. tsutsugamushi* is maintained by transovarial transmission within the population of trombiculid mites. (*1*)

**Fig. 1:**
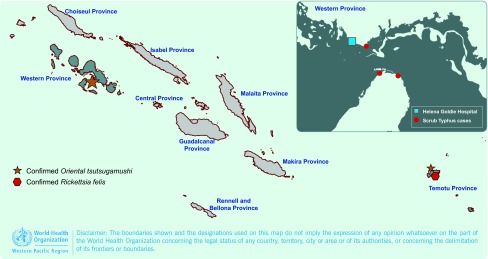
Rickettsial infections in Solomon Islands

***Note:*** The main map shows the proven cases of rickettsial infection in Solomon Islands from 1945 onwards. The inlay map shows the distribution of cases in the current 2014 outbreak.

From 5 to 11 May 2014 there were nine admitted cases of an undiagnosed acute febrile illness at one hospital in Munda, Western Province of Solomon Islands. The cases tested negative on routine microscopy for malaria. These cases were from three villages, namely Dunde, Agagana and Rendova Harbour (**Fig. 1**). Staff from the World Health Organization (WHO) Representative office in Solomon Islands were invited to review thee admitted cases. Finding of an eschar on examination of some of the cases suggested that rickettsial or related infections may be the disease etiology. As a high number of healthy patients having acute fever requiring hospitalization within a short period of time and in such confined areas is unusual, an outbreak investigation was conducted to reveal the etiology and associated risk factors of the illness.

## Methods

An outbreak investigation team consisting of a clinician from Honiara, a clinician from the study hospital, a WHO epidemiologist and staff from the Ministry of Health surveillance unit was formed. The team reviewed routine medical records to obtain demographics, clinical features and treatment outcomes for all suspected cases. A clinically suspected case of rickettsial or related infections was defined as an inpatient in the study hospital with high fever (> 38.5 °C) and a negative malaria microscopy test with an admission date between 1 and 31 May 2014.

The team visited communities from which cases arose between 15 and 16 June 2015. The purpose of the investigation was discussed with village chiefs who assisted in case identification and finding asymptomatic community volunteers as controls for analysis. In each community, the team attempted to locate the cases and recruit two residentially matched controls for each case. All subjects were interviewed using a standardized questionnaire developed by the investigation team, including information on clinical features, risk factors, animal exposure and treatment.

Serum samples were collected from the subjects by venipuncture for testing. Collected samples were transferred to Honiara within 24 hours and were cryopreserved at −80 °C at the National Referral Hospital for later testing. For rickettsial confirmation, serum samples were shipped at room temperature and were in transit for 72 hours before their arrival at the Australian Rickettsial Reference Laboratory that has accreditation for performing rickettsial diagnostics. (*2*) Samples were tested by indirect micro-immunofluorescence assay for total antibodies against six members of the spotted fever group (SFG) rickettsia (including *Rickettsia australis*, *R. honei*, *R. felis*, *R. conorii*, *R. africae* and *R. rickettsii*); typhus group (TG) rickettsia (*R. prowazekii* and *R. typhi*); and scrub typhus (ST) (*Orientia tsutsugamushi*, including Gilliam, Karp and Kato strains). The assay has been described previously. (*3*)

Descriptive analysis was conducted in Excel (Microsoft Excel, Redmond, WA, USA). All individuals from whom samples were collected provided informed written consent which was obtained in local dialect.

## Results

Nine suspected cases were identified by reviewing routine medical records. All cases were male. The median age of the cases was 25 years (interquartile range 18–41 years) with one aged 11 years. The mean duration from symptom onset to hospital admission was six days. All cases presented with an undifferentiated fever syndrome. An eschar, frequently in the groin area, was noted on examination in five cases (55%), and one case developed clinically significant pneumonitis (**Table 1**).

**Table 1:** Clinical symptoms presented in the nine suspected cases for rickettsial infections, Solomon Islands, 2014

Clinical symptoms	*n*(%)
Fever (body temperature > 38.5 °C)	9 (100)
Myalgia	8 (89)
Lymphadenopathy	8 (89)
Headache	5 (56)
Cough	5 (56)
Eschar	5 (56)
Rash	2 (22)

Eight cases were treated with doxycycline and one case was treated with chloramphenicol. Defervescence was reported to occur rapidly following treatment in all cases. Three cases reported treatment with Coartem (Artemether-Lumefantrine) at local clinics before treatment at the hospital. The outbreak investigation team was able to follow up five of the nine cases (55%) and recruit 10 controls (median age 38.5 years, 90% male) for these five cases. Clinical features and demographics did not differ between the follow-up cases and those who were lost to follow-up.

All five cases and 10 controls reported that animals, including rats, were present in both their houses and gardens. There were no reported differences between the cases and controls in the habit of sleeping on the floor, use of mosquito nets and spending time in the bush. Serum samples were obtained from the cases at a median of two weeks following presentation to the hospital or three weeks following the onset of the febrile illness. All five cases (100%) had strong-positive IgG responses to ST (titre ≥ 1:512) which were consistent with a recent acute infection and were considered as confirmed cases of ST. One control (10%) had a moderate-strong total antibody response against ST (titre 1:256). Four controls (40%) had low levels of total antibodies against SFG rickettsia (mean titre 1:128) and one control had a low-level antibody response to TG rickettsia (titre 1:128), suggesting past exposure.

## Discussion

To our knowledge, these are the first laboratory-confirmed cases of ST identified in the Western Province of Solomon Islands. There have been previous laboratory-confirmed cases of both ST and SFG (*R. felis*) in Temotu Province; however Temotu is almost 1000 km across the ocean from Western Province (**Fig. 1**). (*4*, *5*) We found some clinically suspected ST cases that were reported in United States of America soldiers during World War II in ‘North Solomons’ which might refer to Bougainville in Papua New Guinea or some regions of Solomon Islands. (*6*)

All nine ST cases in this study were male. It is unclear if this reflects gender differences regarding health-care access. The presence of eschar is pathognomonic of infection with a rickettsia, but this frequently may not be present. In this study, four cases (44%) did not have a documented eschar, including three of the five laboratory-confirmed cases. It is difficult to distinguish ST and other rickettsia from other causes of undifferentiated fever syndrome when eschars are absent.

Untreated ST has a case fatality ratio of more than 10%, but the disease normally responds well to treatment with doxycycline. (*7*) All cases responded clinically to doxycycline, providing evidence to support our diagnosis.

Solomon Islands Ministry of Health and Medical Services began conducting mass community treatment with azithromycin as part of a trachoma elimination programme shortly after this outbreak began, (*8*) which might have prevented further ST disease transmission in the community.

Rickettsial infections can be confirmed by polymerase chain reaction tests using blood or eschar sample in the acute phase of the disease or by serological methods to detect the rise of antibody titres against ST strains between acute and convalescent serum samples. (*7*) As samples for the acute phase were not available in this investigation, we were unable to perform the laboratory tests above. However, the typical clinical profile (including the presence of eschars) and the very high antibody titres confirmed that ST was the etiology. One control had a low-level antibody response to TG rickettsia, but this was most likely a cross-false positive as this control also had higher titres to the SFG antibodies.

The proportion of malaria that causes fever has been declining in some parts of Solomon Islands from 2008 to 2013. (*9*) Studies in nearby countries including West Papua, Indonesia have shown rickettsia infections are a common cause of acute infections that lead to hospitalization. (*10*) Results of our study may give some insights for the incidence of rickettsia infections in Solomon Islands; however, in the absence of routine testing, the proportion of rickettsial infections that causes febrile illnesses in Solomon Islands is still unclear. A seroprevalence study for rickettsia infections is recommended. This may serve to estimate the incidence of rickettsia infections to help inform management of cases with undifferentiated fever syndromes.

As one of the study limitations, we were unable to obtain serum samples from four of the nine suspected cases; however, the presence of eschars in three of them, along with the results obtained from the other confirmed cases, suggested that the illness in these four cases was also due to ST. Given the small sample size in our study, it is difficult extrapolate these results to the wider population. Statistical analysis was also limited by case numbers. Further studies are recommended to confirm our findings.

In response to this outbreak, the clinical case definition for rickettsial infections was revised to “acute onset of fever (body temperature >38.5 °C) and having eschar OR having malaria microscopy test negative and two or more of the following: lymphadenopathy, headache, myalgia, rash or red eyes” (**Box 1**). Treatment guidelines for rickettsial infections were also developed. Solomon Islands Ministry of Health and Medical Services shared this information with provincial health staff throughout the country for combating future outbreaks of a similar kind.

Box 1 **Revised clinical case definition for rickettsial infections recommended by the Ministry of Health, Solomon Islands, June 2014**

**Table:** 

**Acute onset of fever (body temperature > 38.5 °C) AND either A or B**	
*Group A*	Eschar
*Group B*	Malaria microscopy test negative AND two or more of the following:
	Lymphadenopathy
	Headache
	Myalgia
	Rash
	Red eyes
